# Effects of Stocking Density and Illuminance in Lairage of Fattening Pigs in Different Temperatures

**DOI:** 10.3390/ani14152145

**Published:** 2024-07-23

**Authors:** Dong-Cheol Song, Ji-Hwan Lee, Won Yun, Se-Yeon Chang, Se-Hyun Park, Kyeong-Ho Jeon, Hyuck Kim, Jin-Ho Cho

**Affiliations:** 1 Department of Animal Sciences, Chungbuk National University, Cheongju 28644, Republic of Korea; paul741@daum.net (D.-C.S.); angella2425@naver.com (S.-Y.C.); jeonkh1222@gmail.com (K.-H.J.); harrck85@naver.com (H.K.); 2 Swine Science Division, National Institute of Animal Science, Rural Development Administration, Cheonan 31000, Republic of Korea; junenet123@naver.com; 3 Central Research Institute, Woosung Feed Co., Ltd., Daejeon 34379, Republic of Korea; wyoun@woosung.kr

**Keywords:** stocking density, illuminance, lairage, pig, welfare

## Abstract

**Simple Summary:**

Concerns about animal welfare practices have increased as consumer awareness and regulatory standards have evolved. This study investigated the effects of lairage conditions on welfare and meat quality of pigs during lairage periods. A total of 3070 finishing pigs were assigned to one of six groups arranged in two trials in a 2 × 3 factorial design according to the illuminance (under 40 lux (LX), over 40 lux (HX)) and stocking density (low density (LD), higher than 0.83 m^2^/100 kg; normal density (ND), 0.50–0.83 m^2^/100 kg; high density (HD), lower than 0.50 m^2^/100 kg) with high temperature (HT), higher than 24 °C; low temperature (LT), lower than 10 °C. Based on the obtained results, stocking of too-high (lower than 0.50 m^2^/100 kg) density at HT and stocking of too-low (higher than 0.83 m^2^/100 kg) density at LT are generally not good for meat quality and animal welfare.

**Abstract:**

This study investigated the effects of lairage conditions on the welfare and meat quality of pigs during lairage periods. A total of 3070 finishing pigs were assigned to one of six groups arranged in two trials in a 2 × 3 factorial design according to the illuminance (under 40 lux (LX), over 40 lux (HX)) and stocking density (low density (LD), higher than 0.83 m^2^/100 kg; normal density (ND), 0.50–0.83 m^2^/100 kg; high density (HD), lower than 0.50 m^2^/100 kg) with high temperature (HT), higher than 24 °C; low temperature (LT), lower than 10 °C. Pigs stocked with HD showed lower aggression behavior and overlap behavior than those stocked with LD at LT. Pigs stocked with HD showed higher standing, sitting, and aggression behavior than those stocked with LD at HT. Pigs stocked with HD showed higher pH than those stocked with LD at LT. At HT, pigs stocked with LD showed higher pH, WHC, DL, and CL than those stocked with HD. At LT, pigs stocked with LD showed higher cortisol levels than those stocked with HD. However, pigs stocked with LD showed lower cortisol levels than those stocked with HD at HT. Based on the obtained results, stocking of too-high (lower than 0.50 m^2^/100 kg) density at HT and stocking of too-low (higher than 0.83 m^2^/100 kg) density at LT are generally not good for meat quality and animal welfare.

## 1. Introduction

Generally, pigs are housed in pens before being slaughtered. Keeping familiar pigs in groups and ensuring adequate space in lairage pens are essential prerequisites for ensuring rest and minimizing aversive behavior in lairage. Lairage conditions, including stocking density, illuminance, and ambient temperature, can significantly influence the welfare, physiological responses, and meat quality of fattening pigs during the preslaughter phase [1,2,3]. Concerns about animal welfare practices have increased as consumer awareness and regulatory standards have evolved [4].

Stocking density, defined as the space allowance per pig within a holding area, directly affects animal stress levels and behavior [5]. High density often lead to increased aggression, stress, and subsequent physiological strain, which can compromise immune function and meat quality [6,7].

Illuminance, or the intensity of light exposure, also plays a crucial role in the behavior and stress management of livestock [8]. Proper lighting can reduce fear and injuries by improving visibility, whereas inappropriate lighting can lead to heightened stress and disruptive behaviors [9].

Temperature is a fundamental environmental factor that can affect animal comfort and physiological stability [10]. Extremes of temperature, be it high or low, can exacerbate the negative impacts of unsuitable stocking densities or inappropriate lighting conditions [11]. Thermoregulatory behavior of pigs can be significantly influenced by the thermal environment, which in turn affects their overall welfare and the quality of the pork produced.

Therefore, the objective of this study was to investigate physiological responses and meat quality changes according to lairage stocking density and illuminance in winter and summer.

## 2. Materials and Methods

### 2.1. Ethics

The protocol for this study was reviewed and approved by the Institutional Animal Care and Use Committee of Chungbuk National University, Cheongju, Republic of Korea (approval no. CBNUA-2185-23-02).

### 2.2. Animals, Preslaughter Conditions and Treatments

Between January 2023 and December 2023, a total of 3070 crossbred pigs of mixed sex with the same genetics ((Yorkshire × Landrace) × Duroc) were transported from the commercial finishing farms to the commercial slaughterhouse. A total of 3070 finishing pigs (ibw: 112.76 ± 4.33 kg) were randomly assigned to one of six groups arranged in a 2 × 3 factorial design according to the illuminance by [12] (under 40 lux (LX), over 40 lux (HX)), and stocking density by [13] (low density (LD), higher than 0.83 m^2^/100 kg; normal density (ND), 0.50–0.83 m^2^/100 kg; high density (HD), lower than 0.50 m^2^/100 kg). The experiment was carried out at two different temperatures (high temperature (HT), higher than 24 °C in summer; low temperature (LT), lower than 10 °C in winter). This design was proposed emphasizing the control of all the factors associated with the experimental treatment (genotype, fasting, handling, bedding, distance, and lairage) in order to compare only the effects of lairage density and illuminance.

### 2.3. Meat Quality Measurements

Pig carcasses were graded with the Korean Pig Carcass Grade System. The conductor grades are as follows: 1+ grade (carcass weight: 83 to 93 kg, backfat thickness: 17 to 25 mm), 1 grade (carcass weight: 80 to 98 kg, backfat thickness: 15 to 28 mm), 2 grade (ranges of carcass weight and backfat thickness that do not correspond to 1, 1+ grade). The hot carcass weight was measured on an electronic scale 45 min postmortem and expressed in integer kg units. The left half carcass was used to measure the backfat thickness. The backfat thickness between the last thoracic vertebra and the first lumbar vertebra and that between the 11th and 12th thoracic vertebrae were measured with a ruler. Hot carcass weight and backfat thickness were measured and calculated as follows: (backfat thickness (mm)/hot carcass weight (kg)). Pig losses were measured by observing and classifying fractures and bruises after the pigs were unloaded after transport.

The moisture, protein, and fat content (%) was determined according to the Association of Official Analytical Chemists [14]. The pH was measured after adding 50 mL of distilled water to 5 g of the left carcass loin. All samples were homogenized for 30 s using a homogenizer (Stomacher^®^ 400 Circulator, Seward, UK), and then measured with a pH meter (Orion Star™ A211 pH Benchtop Meter, Thermo scientific™, Cleveland, OH, USA) calibrated in phosphate buffer at pH of 4, 7, and 10. For meat color, the left carcass loin was measured with a Spectro Colorimeter (Model JX-777, Color Techno. System Co., Tokyo, Japan) standardized on a white plate (L*, 89.39; a*, 0.13; b*, −0.51). At this time, the light source used was a white fluorescent lamp (D65). Color values were expressed as L*(lightness), a*(redness), and b*(yellowness). Drip loss (DL) was assessed using the filter paper wetness (FPW) test. Cooking loss (CL) was determined with Oliveira et al.’s (2014) [15] methodology. CL value was measured as the ratio (%) of the weight of the initial sample to the weight after heating the sample. Sensory color was evaluated by 5 trained panelists. The sensory color was as follows: score 1 (pale), score 2 (grayish pink), score 3 (reddish pink), score 4 (purplish red), score 5 (dark). Marbling was evaluated by 5 panelists according to the detailed criteria for grading of livestock products. Marbling score was as follows: score 1 (practically devoid), score 2 (slight), score 3 (modest), score 4 (slightly abundant), score 5 (abundant).

### 2.4. Pork Quality Classes Measurements

The intra-measurement coefficients of variation for meat quality parameters were below 10%. Pork quality classes (pale, soft, and exudative—PSE; red, soft, and exudative -RSE; red, firm, and nonexudative—RFN; pale, firm, and nonexudative—PFN; dark, firm, and dry—DFD) were determined using pH values measured 24 h postmortem, DL variations, and light reflectance (L*), according to [16].

### 2.5. Behavioral and Status Observations and Blood Profile

During lairage, behaviors were continuously recorded using cameras (Intelbras VMH 1010 D HD 720p, Intelbras SA, São José, Brazil), installed on the ceiling of the lairage. During lairage, the number of pigs in each posture (lying, standing, sitting, aggression, and overlap) was recorded. Cortisol, lactate, and glucose samples were taken after 4 h arrival at the lairage. Blood samples were collected from 10 pigs in each group for the determination of concentration levels of cortisol, glucose, and lactate. At least 3 mL of blood samples were taken from the jugular vein. After collection, serum samples were centrifuged at 3000× *g* for 20 min at 4 °C. Thereafter, the blood sample tubes were stored in a −20 °C refrigerator until analysis. The cortisol values were measured using radioimmunoassay Coat-A-Count cortisol kits (Catalog number-TKCO5, Siemens Medical Solution Diagnostics, Los Anglos, CA, USA). Serum glucose was analyzed using an automatic Konelab analyser (Thermo Clinical Labsystems Oy, Vantaa, Finland) according to the manufacturer’s instructions. Lactate levels were measured using a GM7 Analox analyzer (Analox Instruments, London, UK).

### 2.6. Skin Lesion Scoring

Skin lesion scoring was performed for each pig on the ears, shoulders, tails, and total body. Only pigs without skin lesions during the farming and transporting were selected and marked, and skin lesions were measured in lairage by a four-point scale from 0 to 3 [17]. Score 0 was given for a body part if no scratches were found. Score 1 was given if fewer than five superficial scratches were observed, and Score 2 was given if five to ten superficial scratches or fewer than five deep scratches were detected. Score 3 described a body area with more than ten superficial scratches or more than five deep scratches.

### 2.7. Statistical Analysis

Data generated were subjected to a two-way analysis of variance using JMP pro 16.0 software (Statistical Analysis System Software, 2012). Statistics for each factor were analyzed using general linear model (GLM) procedures of SAS. Significantly (*p* < 0.05) different means among the variables were separated using Tukey’s multiple range test, as contained in the same statistical package. Pork quality classes proportion data were analyzed by the nonparametric Kruskal–Wallis test as the data were not normally distributed.

## 3. Results

### 3.1. Behavior Observation

Table 1 shows the effects of lairage stocking density and illuminance on behavior at LT. Pigs stocked with HD showed lower aggression behavior and overlap behavior than those stocked with LD at LT. Also, pigs stocked with LX tended to exhibit higher lying behavior than those stocked with HX at LT. Table 2 shows the effects of lairage stocking density and illuminance on behavior at HT. Pigs stocked with HD showed higher standing, sitting, and aggression behavior than those stocked with LD at HT. However, pigs stocked with LD showed higher lying behavior than those stocked with HD at HT. There was no interaction between stocking density and illuminance.

### 3.2. Carcass Grade and Carcass Composition

Table 3 and Table 4 show the effects of lairage stocking density and illuminance on carcass grade and composition at LT and HT, respectively. At HT, pigs stocked with LD showed lower backfat thickness than those stocked with HD. Also, pigs stocked at LD and HD showed numerically higher 1+ grade than those stocked at HD and LD at HT and LT, respectively. There was no interaction between stocking density and illuminance.

### 3.3. Pork Quality

Table 5 and Table 6 show the effects of lairage stocking density and illuminance on pork quality at LT and HT, respectively. Pigs stocked with HD showed higher pH than those stocked with LD at LT. At HT, pigs stocked with LD showed higher pH, WHC, DL, and CL than those stocked with HD. There was no interaction between stocking density and illuminance.

### 3.4. Blood Profile

Table 7 and Table 8 show the effects of lairage stocking density and illuminance on blood profile at LT and HT, respectively. At LT, pigs stocked with LD showed higher cortisol level than those stocked with HD. However, pigs stocked with LD showed lower cortisol level than those stocked with HD at HT. There was no interaction between stocking density and illuminance.

### 3.5. Skin Lesion Score

Table 9 and Table 10 show the effects of lairage stocking density and illuminance on skin lesion score at LT and HT, respectively. At LT, pigs stocked at LD showed numerically higher skin lesion (shoulder and body) than those stocked at HD. Under HT condition, pigs stocked at HD showed numerically higher skin lesion (ear and body) than those stocked at LD.

## 4. Discussion

Observed behavioral differences between pigs stocked at LD and HD indicated that pig behavior was sensitive to environmental conditions. In the LT condition, HD led to reduced aggression and overlap behaviors, indicating a potential decrease in stress levels among pigs. According to [18], low stocking density in lairage can lead to cold stress. Conversely, in the HT condition, a contrasting behavioral response was observed, with HD-stocked pigs displaying higher levels of standing, sitting, and aggression behavior than LD-stocked pigs. Increased activity and aggression among HD-stocked pigs at HT might be attributed to heat stress (HS) and heightened competition for resources, such as space and ventilation [19]. The HS can exacerbate behavioral responses, leading to restlessness and aggression as pigs seek relief from thermal discomfort [20]. Observed increases of standing and sitting behaviors might reflect efforts to dissipate heat and regulate body temperature, while heightened aggression may stem from increased frustration and irritability in crowded conditions [21]. Lying behavior serves as a crucial indicator of pig welfare as it reflects the animals’ ability to rest and recuperate in lairage environment [7]. Interestingly, pigs stocked at LD at HT exhibited higher levels of lying behavior than HD-stocked pigs. Illuminance is another crucial factor that can significantly impact pig behavior, welfare, and overall productivity. Pigs are known to be sensitive to changes in light conditions, which can influence their physiological and psychological states. Appropriate lighting conditions can help reduce stress and aggression among pigs, promote more regular feeding and resting patterns, and enhance overall wellbeing [22]. In this study, pigs stocked with LX showed high tendency of lying behavior than those stocked with HX at LT. According to these findings, stocking density impacts behavior differently depending on temperature conditions, emphasizing the importance of adaptive management practices.

In HT conditions, pigs stocked at LD exhibited lower backfat thickness than those stocked at HD. This finding suggests that lower stocking densities may contribute to leaner carcasses, potentially due to increased physical activity and reduced adipose tissue deposition in less crowded environments [23,24]. The 1+ grade typically indicates superior meat quality characteristics, such as higher marbling and tenderness, which are desirable attributes in the pork industry [25,26]. The numerically higher prevalence of 1+ grade in LD-stocked pigs suggests potential benefits associated with lower stocking densities, including improved meat quality and carcass characteristics. This finding suggests that HS might influence adipose tissue deposition, potentially impacting carcass composition and yield [27]. However, further research is needed to elucidate the underlying physiological mechanisms driving these differences and to assess their implications for overall meat quality.

pH is a critical indicator of meat quality. Higher pH values are often associated with reduced acidity and improved meat tenderness and juiciness [28]. The observed increase in pH among HD-stocked pigs suggests potential differences in muscle metabolism and postmortem processes related to stocking density. Conversely, in HT conditions, pigs stocked at LD displayed superior pork quality characteristics to HD-stocked pigs. LD-stocked pigs exhibited higher pH, WHC, DL, and CL, indicating improved water retention and reduced cooking losses. Improved WHC, DL, and CL reflect better moisture retention and cooking properties, which are desirable traits of pork products [29]. These temperature-dependent effects underscore the importance of considering environmental conditions when evaluating pork quality parameters and implementing management strategies to optimize product attributes.

In LT conditions, pigs stocked at LD exhibited higher cortisol levels than those stocked at HD. Elevated cortisol levels indicate increased stress and arousal in pigs, which may result from factors such as crowding, competition for resources, and environmental discomfort [30]. Higher cortisol levels observed in LD-stocked pigs suggest that higher stocking densities may exacerbate stress levels and welfare concerns in pigs exposed to cooler temperatures during lairage. Conversely, in HT conditions, a contrasting pattern was observed, with LD-stocked pigs displaying lower cortisol levels than HD-stocked pigs. This unexpected finding suggests that lower stocking densities may mitigate stress responses in pigs exposed to higher temperatures during lairage. HS can exacerbate physiological stress responses, including cortisol secretion, as animals struggle to thermoregulate and cope with thermal discomfort [20]. Lower cortisol levels observed in LD-stocked pigs at HT may indicate reduced stress and improved welfare under less crowded conditions.

Skin damage can act as an animal welfare indicator and reflect the quality of the animal’s social and physical environment [31]. Unadapted social environment can cause excessive fighting (e.g., after mixing), ultimately resulting in skin damage [32]. The higher prevalence of skin lesions observed in LD-stocked pigs at LT might be attributed to increased competition for resources and space within the lairage environment. Lower stocking densities may afford pigs more room to move and rest comfortably, potentially reducing the likelihood of skin abrasions and injuries [11]. In contrast, numerically higher skin lesions observed in HD-stocked pigs at HT suggest that HS and overcrowding may contribute to increased skin injuries in these animals. High ambient temperatures can exacerbate stress levels and discomfort among pigs, leading to heightened agitation and aggression, which may result in skin abrasions and lesions [33]. Additionally, reduced space availability in high-density stocking scenarios may exacerbate social stress and competition, further increasing the likelihood of skin injuries.

Overall, the findings of this study underscore the complexity of interactions between lairage stocking density, illuminance, and temperature conditions, and their effects on pig behavior, carcass quality, pork quality, blood profile, and skin lesion scores. Adopting integrated management approaches that consider these interactions is essential for promoting both animal welfare and meat quality in pig production systems. Further research is needed to elucidate the underlying mechanisms driving these effects and develop targeted interventions to optimize pig welfare and product quality in diverse production environments.

## 5. Conclusions

Pigs exposed to HD (lower than 0.5 m^2^/100 kg) at HT during preslaughter caused acute stress. Also, pigs exposed to LD (lower than 0.5 m^2^/100 kg) at LT during preslaughter caused acute stress. Based on the obtained results, stocking of too-high (lower than 0.50 m^2^/100 kg) density at HT and stocking of too-low (higher than 0.83 m^2^/100 kg) density at LT are generally not good for meat quality and animal welfare. Therefore, higher stocking density (lower than 0.50 m^2^/100 kg) at LT (lower than 10 °C in winter) and lower stocking (higher than 0.83 m^2^/100 kg) density at HT (higher than 24 °C in summer) are recommended.

## Figures and Tables

**Table 1:** Effects of lairage stocking density and illuminance on behaviors at low temperature.

Items	Main Effect					SE	*p*-Value		
	D			IL					
	HD	ND	LD	LX	HX		D	IL	D × IL
Standing	38.90	36.56	34.73	34.34	39.12	3.495	0.485	0.122	0.077
Sitting	6.43	5.55	6.20	5.61	6.51	0.068	0.289	0.129	0.095
Lying	14.67	17.89	19.07	20.05	14.38	3.612	0.397	0.078	0.179
Aggression	3.19 b	3.59 ab	4.05 a	3.6	3.62	0.212	0.002	0.919	0.583
Overlap	1.26 b	1.55 ab	2.30 a	1.63	1.77	0.348	0.037	0.652	0.696

Abbreviation: D, density; IL, illuminance; D × IL, density × illuminance; SE, standard error. HD (lower than 0.50 m^2^/100 kg); ND (0.50 m^2^~0.83 m^2^/100 kg); LD (higher than 0.83 m^2^/100 kg). LX, low illuminance; HX, high illuminance. Standing, the act of standing still without any other action, with the forelimbs and hind legs stretched perpendicularly to the floor or similar behavior; sitting, two front legs straight to the floor, two rear legs and hips sitting in contact with the floor or similar behavior; lying, the act of lying in the most comfortable position with the head, front legs, back legs, and abdomen touching the floor or similar behavior; aggression, pushing, biting, or beating another pig with the head, lifting the pigs by pushing the head under the body or similar behavior; overlap, the act of placing both forelimbs on the back of another pig or similar behavior. a, b: means in the same row with different superscripts differ (*p* < 0.05).

**Table 2:** Effects of lairage stocking density and illuminance on behaviors at high temperature.

Items	Main Effect					SE	*p*-Value		
	D			IL					
	HD	ND	LD	LX	HX		D	IL	D × IL
Standing	36.32 a	32.09 ab	28.83 b	31.17	33.65	2.558	0.044	0.253	0.478
Sitting	5.79 a	5.19 ab	4.33 b	4.99	5.21	0.484	0.034	0.573	0.559
Lying	17.89 b	22.73 ab	26.85 a	23.84	21.14	2.467	0.011	0.197	0.552
Aggression	0.87 a	0.72 ab	0.60 b	0.75	0.71	0.062	0.002	0.373	0.351
Overlap	0.53	0.56	0.59	0.52	0.6	0.064	0.707	0.138	0.278

Abbreviation: D, density; IL, illuminance; D × IL, density × illuminance; SE, standard error. HD (lower than 0.50 m^2^/100 kg); ND (0.50 m^2^~0.83 m^2^/100 kg); LD (higher than 0.83 m^2^/100 kg). LX, low illuminance; HX, high illuminance. Standing, the act of standing still without any other action, with the forelimbs and hind legs stretched perpendicularly to the floor or similar behavior; sitting, two front legs straight to the floor, two rear legs and hips sitting in contact with the floor or similar behavior; lying, the act of lying in the most comfortable position with the head, front legs, back legs, and abdomen touching the floor or similar behavior; aggression, pushing, biting, or beating another pig with the head, lifting the pigs by pushing the head under the body or similar behavior; overlap, the act of placing both forelimbs on the back of another pig or similar behavior. a, b: Means in the same row with different superscripts differ (*p* < 0.05).

**Table 3:** Effects of lairage stocking density and illuminance on carcass trait at low temperature.

Items	Main Effect					SE	*p*-Value		
	D			IL					
	HD	ND	LD	LX	HX		D	IL	D × IL
**Carcass composition traits**									
Hot carcass weight, kg	87.58	86.21	85.87	86.42	86.68	0.79	0.11	0.69	0.19
Backfat thickness, mm	24.03	23.61	23.00	23.87	23.23	0.64	0.31	0.23	0.24
**Carcass grade score**									
Grade 1+, %	50.00	40.00	20.00	27.60	32.00	-	0.433	0.588	-
Grade 1, %	26.00	26.90	36.70	29.70	49.20	-	0.769	0.234	-
Grade 2, %	24.00	33.10	43.30	42.70	18.80	-	0.396	0.221	-

Abbreviation: D, density; IL, illuminance; D × IL, density × illuminance; SE, standard error. HD (lower than 0.50 m^2^/100 kg); ND (0.50 m^2^~0.83 m^2^/100 kg); LD (higher than 0.83 m^2^/100 kg). LX, low illuminance; HX, high illuminance; carcass grade score was measured by Korean Ministry of Agriculture, Food and Rural Affairs Notification. Grade 1+, 83 kg~93 kg of carcass weight with 17–25 mm of backfat thickness; Grade 1, 80 kg~98 kg of carcass weight with 15–28 mm of backfat thickness; Grade 2, pigs do not belong to Grade 1+ and Grade 1.

**Table 4:** Effects of lairage stocking density and illuminance on carcass trait at high temperature.

Items	Main Effect					SEM	*p*-Value		
	D			IL					
	HD	ND	HD	LX	HX		D	IL	D × IL
**Carcass composition traits**									
Hot carcass weight, kg	88.20	89.05	88.17	88.31	88.64	0.45	0.11	0.38	0.35
Backfat thickness, mm	24.70 a	24.45 ab	23.53 b	24.14	24.31	0.42	0.02	0.62	0.82
**Carcass grade score**									
Grade 1+, %	31.00	39.00	41.00	36.00	36.00	-	0.336	0.883	-
Grade 1, %	31.00	36.00	36.00	29.00	37.00	-	0.267	0.644	-
Grade 2, %	38.00	25.00	23.00	35.00	37.00	-	0.459	0.311	-

Abbreviation: D, density; IL, illuminance; D × IL, density × illuminance; SEM, standard error of means. HD (lower than 0.50 m^2^/100 kg); ND (0.50 m^2^~0.83 m^2^/100 kg); LD (higher than 0.83 m^2^/100 kg). LX, low illuminance; HX, high illuminance; carcass grade score was measured by Korean Ministry of Agriculture, Food and Rural Affairs Notification. Grade 1+, 83 kg~93 kg of carcass weight with 17–25 mm of backfat thickness; Grade 1, 80 kg~98 kg of carcass weight with 15–28 mm of backfat thickness; Grade 2, pigs do not belong to Grade 1+ and Grade 1. a, b: means in the same row with different superscripts differ (*p* < 0.05).

**Table 5:** Effects of lairage stocking density and illuminance on pork quality at low temperature.

Items	Main Effect					SE	*p*-Value		
	D			IL			D	IL	D × IL
	HD	ND	LD	LX	HX				
**Pork composition, %**									
Moisture	73.15	73.49	73.87	73.76	73.25	0.597	0.508	0.312	0.684
Crude protein	22.82	23.08	21.81	22.26	22.88	1.072	0.483	0.495	0.716
Crude fat	2.24	2.56	1.63	2.18	2.11	0.645	0.372	0.892	0.898
**Pork quality parameters**									
pH	5.59 a	5.50 b	5.50 b	5.52	5.54	0.034	0.031	0.520	0.72
WHC, %	63.87	60.26	60.9	60.46	62.89	3.696	0.595	0.437	0.613
DL, %	4.44	4.53	4.74	4.58	4.57	0.19	0.302	0.928	0.941
CL, %	24.43	23.19	26.22	25.11	24.11	2.621	0.527	0.649	0.924
L* value	49.25	49.77	50.62	50.54	49.22	2.543	0.864	0.536	0.715
a* value	7.65	6.48	5.42	6.71	6.32	0.863	0.071	0.583	0.402
b* value	5.89	5.62	4.8	5.63	5.24	0.443	0.072	0.299	0.358
sensory color ^1)^	2.83	2.67	2.5	2.72	2.61	0.366	0.67	0.717	0.409
Marbling ^2)^	3.15	3.15	3.23	3.12	3.23	0.71	0.991	0.851	0.889
**Pork quality classes (%)**									
PSE pork	10	15	30	20	16.67	-	0.215	0.321	-
RSE pork	10	10	10	10	10	-	0.684	0.745	-
RFN pork	40	10	50	43.33	43.33	-	0.573	0.198	-
PFN pork	40	35	10	26.67	30	-	0.742	0.138	-
DFD pork	0	0	0	0	0	-	1	1	-

Abbreviation: D, density; IL, illuminance; D × IL, density × illuminance; SE, standard error. HD (lower than 0.50 m^2^/100 kg); ND (0.50 m^2^~0.83 m^2^/100 kg); LD (higher than 0.83 m^2^/100 kg). LX, low illuminance; HX, high illuminance. ^1)^ Color score ranged from 1 (pale color) to 5 (dark color). ^2)^ Marbling score ranged from 1 (practically devoid) to 5 (abundant). WHC, water-holding capacity; DL, drip loss; CL, cooking loss; PSE, pale, soft, exudative; RSE, reddish–pink, soft, exudative; RFN, red, firm, nonexudative; PFN, pale, firm, nonexudative; DFD, dark, firm, dry.

**Table 6:** Effects of lairage stocking density and illuminance on pork quality at high temperature.

Items	Main Effect					SE	*p*-Value		
	D			IL			D	IL	D× IL
	HD	ND	LD	LX	HX				
**Pork composition, %**									
Moisture	72.41 b	73.38 ab	74.07 a	73.26	73.30	0.489	0.017	0.921	0.443
Crude protein	23.02	22.80	22.49	23.13	22.42	0.919	0.847	0.365	0.899
Crude fat	2.13	2.07	2.01	2.33	2.23	0.660	0.553	0.852	0.723
**Pork quality parameters**									
pH	5.46 b	5.55 ab	5.69 a	5.57	5.56	0.070	0.021	0.859	0.469
WHC, %	63.66 b	67.02 ab	69.70 a	67.37	66.22	1.821	0.020	0.456	0.855
DL, %	5.00 a	4.80 ab	4.42 b	4.78	4.71	0.164	0.013	0.599	0.452
CL, %	25.26 a	23.25 b	22.98 b	23.57	24.09	0.716	0.015	0.389	0.995
L* value	51.58	51.21	49.57	50.02	51.55	1.987	0.577	0.365	0.608
a* value	7.08	6.72	5.78	6.50	6.56	0.795	0.280	0.923	0.395
b* value	6.04	5.32	4.65	5.33	5.35	0.629	0.128	0.976	0.160
sensory color	2.83	2.75	2.92	2.78	2.89	0.495	0.945	0.788	0.517
Marbling	3.08	3.22	3.15	3.30	3.00	0.300	0.907	0.244	0.843
**Pork quality classes (%)**									
PSE pork	50.00	16.67	0.00	22.22	22.22		0.368	0.121	
RSE pork	16.67	16.67	0.00	11.11	11.11		0.684	0.105	
RFN pork	0.00	33.33	83.33	55.56	22.22		0.073	0.098	
PFN pork	33.33	33.33	16.67	11.11	44.45		0.842	0.138	
DFD pork	0.00	0.00	0.00	0.00	0.00		1	1	

Abbreviation: D, density; IL, illuminance; D × IL, density × illuminance; SE, standard error. HD (lower than 0.50 m^2^/100 kg); ND (0.50 m^2^~0.83 m^2^/100 kg); LD (higher than 0.83 m^2^/100 kg). LX, low illuminance; HX, high illuminance. ^1)^ Color score ranged from 1 (pale color) to 5 (dark color). ^2)^ Marbling score ranged from 1 (practically devoid) to 5 (abundant). WHC, water-holding capacity; DL, drip loss; CL, cooking loss; PSE, pale, soft, exudative; RSE, reddish–pink, soft, exudative; RFN, red, firm, nonexudative; PFN, pale, firm, nonexudative; DFD, dark, firm, dry. a, b: means in the same row with different superscripts differ (*p* < 0.05).

**Table 7:** Effects of lairage stocking density and illuminance on blood profile at low temperature.

Items	Main Effect					SE	*p*-Value		
	D			IL					
	HD	ND	LD	LX	HX		D	IL	D × IL
Cortisol (ug/dL)	3.86 b	3.95 ab	4.05 a	3.93	3.98	0.08	0.048	0.399	0.645
Lactate (mmol/L)	4.66	4.71	4.71	4.68	4.71	0.13	0.892	0.776	0.225
Glucose (mg/dL)	81.60	82.15	82.00	81.73	82.10	1.14	0.883	0.695	0.495

Abbreviation: D, density; IL, illuminance; D × IL, density × illuminance; SE, standard error. HD (lower than 0.50 m^2^/100 kg); ND (0.50 m^2^~0.83 m^2^/100 kg); LD (higher than 0.83 m^2^/100 kg). LX, low illuminance; HX, high illuminance. a, b: means in the same row with different superscripts differ (*p* < 0.05).

**Table 8:** Effects of lairage stocking density and illuminance on blood profile at high temperature.

Items	Main Effect					SE	*p*-Value		
	D			IL					
	HD	ND	LD	LX	HX		D	IL	D × IL
Cortisol (ug/dL)	4.02 a	3.94 ab	3.84 b	3.94	3.93	0.06	0.021	0.876	0.544
Lactate (mmol/L)	4.61	4.51	4.44	4.55	4.49	0.10	0.255	0.499	0.361
Glucose (mg/dL)	78.70	78.80	78.00	78.20	78.80	1.19	0.765	0.539	0.765

Abbreviation: D, density; IL, illuminance; D × IL, density × illuminance; SE, standard error. HD (lower than 0.50 m^2^/100 kg); ND (0.50 m^2^~0.83 m^2^/100 kg); LD (higher than 0.83 m^2^/100 kg). LX, low illuminance; HX, high illuminance. a, b: means in the same row with different superscripts differ (*p* < 0.05).

**Table 9:** Effects of lairage stocking density and illuminance on skin lesion score at low temperature.

Items	Main Effect				
	D			IL	
	HD	ND	LD	LX	HX
**Skin Lesion Score**					
**Ear**					
1	82.22	84.44	80.00	80.00	90.00
2	15.56	11.11	13.33	16.67	10.00
3	2.22	4.44	6.67	3.33	0.00
4	0.00	0.00	0.00	0.00	0.00
**Tail**					
1	77.78	86.67	76.67	83.33	86.67
2	17.78	11.11	23.33	10.00	10.00
3	4.44	2.22	0.00	6.67	3.33
4	0.00	0.00	0.00	0.00	0.00
**Shoulder**					
1	77.78	88.89	80.00	83.33	86.67
2	15.56	4.44	6.67	10.00	13.33
3	6.67	6.67	13.33	6.67	0.00
4	0.00	0.00	0.00	0.00	0.00
**Total body**					
1	88.89	88.89	86.67	86.67	93.33
2	8.89	4.44	3.33	10.00	6.67
3	2.22	6.67	10.00	3.33	0.00
4	0.00	0.00	0.00	0.00	0.00

Abbreviation: D, density; IL, illuminance; D × IL, density × illuminance; HD (lower than 0.50 m^2^/100 kg); ND (0.50 m^2^~0.83 m^2^/100 kg); LD (higher than 0.83 m^2^/100 kg LX, low illuminance; HX, high illuminance; For lesion scoring, a four-point scale from 0 to 3 was used [17]. Score 0 given for a body part if no scratches were found; Score 1 given if fewer than five superficial scratches; Score 2 given if five to ten superficial scratches or fewer than five deep scratches; Score 3 describes a body area with more than ten superficial scratches or more than five deep scratches.

**Table 10:** Effects of lairage stocking density and illuminance on skin lesion score at high temperature.

Items	Main Effect				
	D			IL	
	HD	ND	LD	LX	HX
**Skin Lesion Score**					
**Ear**					
1	76.67	86.67	90.00	84.44	84.44
2	13.33	10.00	10.00	13.33	8.89
3	10.00	3.33	0.00	2.22	6.67
4	0.00	0.00	0.00	0.00	0.00
**Tail**					
1	73.33	76.67	80.00	77.78	75.56
2	20.00	20.00	20.00	17.78	22.22
3	6.67	3.33	0.00	4.44	2.22
4	0.00	0.00	0.00	0.00	0.00
**Shoulder**					
1	86.67	86.67	90.00	88.89	86.67
2	6.67	6.67	10.00	6.67	8.89
3	6.67	6.67	0.00	4.44	4.44
4	0.00	0.00	0.00	0.00	0.00
**Total body**					
1	80.00	86.67	90.00	86.67	84.44
2	10.00	10.00	10.00	11.11	8.89
3	10.00	3.33	0.00	2.22	6.67
4	0.00	0.00	0.00	0.00	0.00

Abbreviation: D, density; IL, illuminance; D × IL, density × illuminance; HD (lower than 0.50 m^2^/100 kg); ND (0.50 m^2^~0.83 m^2^/100 kg); LD (higher than 0.83 m^2^/100 kg). LX, low illuminance; HX, high illuminance. For lesion scoring, a four-point scale from 0 to 3 was used [17]. Score 0 given for a body part if no scratches were found; Score 1 given if fewer than five superficial scratches; Score 2 given if five to ten superficial scratches or fewer than five deep scratches; Score 3 describes a body area with more than ten superficial scratches or more than five deep scratches.

## Data Availability

The original contributions presented in the study are included in the article, further inquiries can be directed to the corresponding author.
